# Attenuating Muscle Damage Biomarkers and Muscle Soreness After an Exercise-Induced Muscle Damage with Branched-Chain Amino Acid (BCAA) Supplementation: A Systematic Review and Meta-analysis with Meta-regression

**DOI:** 10.1186/s40798-024-00686-9

**Published:** 2024-04-16

**Authors:** Atef Salem, Khouloud Ben Maaoui, Haithem Jahrami, Mezna A. AlMarzooqi, Omar Boukhris, Balsam Messai, Cain C. T. Clark, Jordan M. Glenn, Hadeel A. Ghazzaoui, Nicola Luigi Bragazzi, Achraf Ammar, Khaled Trabelsi, Hamdi Chtourou

**Affiliations:** 1 Higher Institute of Sport and Physical Education of Sfax, University of Sfax, 3000 Sfax, Tunisia; 2 Physical Activity, Sport, and Health, UR18JS01, National Observatory of Sport, 1003 Tunis, Tunisia; 3 Ministry of Health, Manama, 410 Bahrain; 4 Department of Psychiatry, College of Medicine and Medical Sciences, Arabian Gulf University, Manama, 323 Bahrain; 5 Leaders Development Institute, Ministry of Sport, Riyadh, Saudi Arabia; 6 Sport and Exercise Science, School of Allied Health, Human Services and Sport, La Trobe University, Melbourne, 3086 Australia; 7 Centre for Intelligent Healthcare, Coventry University, Coventry, CV1 5FB UK; 8 Department of Health, Exercise Science Research Center Human Performance and Recreation, University of Arkansas, Fayetteville, AR 72701 USA; 9 Department Nutrition and Food Technology, School of Agriculture, The University of Jordan, Amman, 11942 Jordan; 10 Laboratory for Industrial and Applied Mathematics (LIAM), Department of Mathematics and Statistics, York University, Toronto, ON M3J 1P3 Canada; 11 Department of Training and Movement Science, Institute of Sport Science, Johannes Gutenberg-University Mainz, Mainz, Germany; 12 Research Laboratory: Education, Motricity, Sport and Health, EM2S, LR19JS01, University of Sfax, 3000 Sfax, Tunisia

**Keywords:** Branched-chain amino acid, Muscle damage, Recovery, Muscle soreness, Creatine kinase, Lactate dehydrogenase

## Abstract

**Background:**

Branched-chain amino acid (BCAA) supplementation is one of the most popular strategies used by the general population and athletes to reduce muscle soreness and accelerate the recovery process of muscle damage biomarkers after an intense exercise or training session.

**Objectives:**

This systematic review and meta-analysis investigated the effects of BCAA supplementation on muscle damage biomarkers and muscle soreness after exercise-induced muscle damage (EIMD).

**Methods:**

The systematic literature search for randomized controlled trials was conducted using seven databases, up to September 13th, 2022. The eligibility criteria for selecting studies were as follows: studies performed on healthy active participants, using BCAA at least once, controlled with a placebo or control group, performing resistance or endurance exercises, and followed up at least once post-EIMD. The methodological quality of the studies was assessed using the “SIGN RCT checklist”. Random-effects meta-analyses were processed to compute the standardized mean difference (Hedges’ g). Meta-regression analyses were completed with daily and total dosage and supplementation as continuous moderator variables.

**Results:**

Of the 18 studies included in this meta-analysis, 13 were of high quality and five were of acceptable quality. Our results revealed BCAA supplementation elicits a significant effect on reducing creatine kinase (CK) levels immediately (g = − 0.44; *p* = 0.006) and 72 h (g = − 0.99; *p* = 0.002), but not 24 h, 48 h, and 96 h post-EIMD. Additionally, a significant effect on delayed onset of muscle soreness (DOMS) was identified at 24 h (g = − 1.34; *p* < 0.001), 48 h (g = − 1.75; *p* < 0.001), 72 h (g = − 1.82; *p* < 0.001), and 96 h (g = − 0.82; *p* = 0.008), but not immediately post-EIMD. No significant effect was found on lactate dehydrogenase (LDH) levels at any time point. Meta-regression indicated higher daily and total dosages of BCAA, and longer supplementation periods were related to the largest beneficial effects on CK (total dosage and supplementation period) at 48 h, and on DOMS at 24 h (only daily dosage).

**Conclusion:**

The overall effects of BCAA supplementation could be considered useful for lowering CK and DOMS after EIMD, but not LDH. The longer supplementation period prior to the EIMD could be more effective for CK and DOMS reduction.

**Supplementary Information:**

The online version contains supplementary material available at 10.1186/s40798-024-00686-9.

## Key Points

- Branched-chain amino acid supplementation is likely to reduce muscle soreness and creatine kinase, but not lactate dehydrogenase.
- The responses of muscle damage biomarkers and muscle soreness to branched-chain amino acid supplementation may be influenced by study design, blinding, training status, and sex.
- Dose-response relationships revealed high daily doses of branched-chain amino acids, with a long supplementation period (i.e., > 7 days), could be more effective in lowering creatine kinase levels.
- A high total dosage of branched-chain amino acids (i.e., > 5 g/day) could be more effective in reducing muscle soreness.

**Supplementary Information:**

The online version contains supplementary material available at 10.1186/s40798-024-00686-9.

## Background

Exercise-included muscle damage (EIMD) is typically induced by eccentric muscle contractions. EIMD reduces physical performance by decreasing maximum muscle strength and range of motion, as well as exacerbating musculoskeletal and neurological problems [1, 2]. Additionally, EIMD causes increased delayed onset muscle soreness (DOMS), intramuscular proteins in the blood (i.e., creatine kinase (CK), lactate dehydrogenase (LDH), and myoglobin), and muscle inflammatory biomarkers, lasting for several days [3–5]. Although these symptoms are highly individualized [6], they frequently peak between 24 and 48 h after the initial bout and are generally healed within 7 days [7]. Moreover, studies have reported an increase in biomarkers of muscle damage (CK and LDH) following repeated sprint exercises [8–13] and resistance training sessions [14], with peaks immediately and 24 h after EIMD.

Branched-chain amino acids (BCAA: leucine, isoleucine, and valine) comprise approximately 50% of essential amino acids (EAAs) in food and 35% of the total content of EAAs in muscle proteins [15, 16]. BCAA components are catabolized first in the skeletal muscles, while other amino acids are catabolized in the liver [15]. BCAA supplementation has been proposed as an alternative dietary strategy for reducing muscle damage and fatigue induced by EIMD. The effects of BCAA on muscle cell regeneration and restoration as a nutrition therapy have gained increased attention in recent years. BCAA could directly regulate protein turnover in muscle cells to reverse the catabolic and anti-anabolic consequences of EIMD [17]. Leucine has been recognized as a crucial regulator of mammalian target of rapamycin signalling and translation [18, 19]. Furthermore, it has been proposed BCAA could potentially play a role as promoters in the recovery process of modified muscle tissues, which are predominantly comprised of proteins [20]. These modified muscle tissues are frequently induced by mechanical strain and inflammation during physical exertion [20]. Within this context, BCAA are widely thought to confer advantageous outcomes by actively aiding the recuperation and restoration of such tissues [21]. As a result of its potential to alleviate the negative symptoms of EIMD, usage of BCAA as a supplementation approach has increased in popularity among sedentary and active individuals and athletes [22].

BCAA are also major precursors of tricarboxylic acid (TCA) cycle intermediates via acetyl-CoA and succinyl-CoA [23]. BCAA can decrease serotonin production in the brain and reduce the onset of central fatigue [24] by influencing the blood level of free tryptophan (fTRP) [25]. It should be noted that valine and fTRP compete for the same transport sites along the blood–brain barrier and the increased BCAA concentrations can lower the fTRP-to-BCAA ratio [25]. It is suggested that higher utilization of BCAA mixtures may be attenuated by increased use of the BCAA aminotransferase process to produce glutamine, which is a crucial step in ammonia detoxification to glutamine in the muscles [21]. Therefore, BCAA supplementation appears to be a helpful strategy for recovery between workouts and may have a positive impact on subsequent exercise performance [24, 26].

The effects of BCAA supplementation on EIMD mitigation and muscle soreness were investigated thoroughly across different exercise conditions and populations, albeit with variations in findings and studies’ methodological qualities [20]. Previous systematic reviews and meta-analyses reveal BCAA supplementation reduces muscle damage biomarkers [27–31] and muscle soreness [27, 29–32]. Nevertheless, the latter reviews were conducted with diverse eligibility criteria with respect to study design, blinding, training status, sex, damaging exercise protocol, or intervention. This diversity in eligibility criteria may have influenced the results of the meta-analyses. It is worth noting that only the study of Khemtong et al. [29] was conducted with restricted criteria by limiting the analysis to male-trained athletes participating in resistance-damaging protocols. Additionally, healthy active participants and studies including endurance-damaging protocols were excluded [29]. Moreover, previous meta-analyses [27, 28, 30, 31] were conducted by including protocols using BCAA supplementation combined with other ingredients such as protein [33], green tea [34], arginine [35, 36] and vitamins (A, E, and B6) [37]. Therefore, the independent effect of BCAA supplementation is impossible to identify. Recently, Kadlec et al. [38] identified common statistical errors in meta‑analyses pertaining to the field of strength and conditioning research. The authors concluded the identified statistical errors impacted the results of the meta-analyses, leading to flawed conclusions. For example, ignoring outliers in meta-analysis processing might have a profound impact on the result's effectiveness and stability [38]. Previous meta-analyses [29, 30] investigating the effect of BCAA ingestion on muscle damage biomarkers and muscle soreness overlooked the influence of diagnostics and sensitivity analyses in order to detect outliers and identify their influence on the overall effect size. Therefore, it seems that a more robust meta-analytical approach should be adopted for a stable overall effect size.

As such, this systematic review and meta-analysis provides an update of the evidence on the effects of BCAA supplementation on muscle damage biomarkers and muscle soreness across multiple follow-up time points after EIMD, as well as identifies dose–response effects of the daily dosage, the total dosage, and the supplementation period of BCAA. We hypothesized BCAA supplementation would decrease CK levels and DOMS post-EIMD, but not LDH levels.

## Methods

### Study Protocol

This systematic review and meta-analysis was conducted and completed following the Preferred Reporting Items for Systematic Reviews and Meta-Analysis (PRISMA) guidelines [39] and the adapted PRISMA guidelines in sport science [40]. Inclusion criteria were chosen using the PICOS model (Population, Intervention, Comparator, Outcomes, and Study design) (Table 1).

**Table 1:** PICOS model used in this meta-analysis

Parameter	Criteria
Population	Healthy active participants
Intervention	BCAA supplementation
Comparator	Placebo supplementation
Outcomes	CK, LDH, DOMS
Study design	Randomized controlled trials

BCAA: Branched-chain amino acids, CK: Creatine kinase, LDH: Lactate dehydrogenase, DOMS: Delayed onset of muscle soreness

### Eligibility Criteria

Only articles and studies meeting all following criteria were included in this meta-analysis: (i) full-text published articles; (ii) randomized controlled trials (parallel or crossover study design); (iii) performed on healthy active participants; *(iv)* using BCAA as an intervention at least once; (v) controlled with placebo intervention or control group; (vi) supplementation pre-EIMD or pre- and post-EIMD for BCAA and placebo interventions; (vii) using resistance or endurance exercises as an EIMD protocol; (viii) follow-up time points at least once after the EIMD. Studies using co-ingestion of other essential amino acids or other ingredients with BCAA were excluded. Additionally, articles using BCAA supplementation for specific disease treatment or medical intervention were excluded.

### Search Strategy

The systematic literature search was conducted using seven online databases (PubMed, Web of Science, Scopus, SPORTDiscus, CINAHL, ProQuest, and OpenGrey), from database inception to September 13th, 2022. Appropriate Boolean operators (AND, OR, and NOT) were used to join the various keywords. Field tags, wild-card options (i.e., truncated words), and medical subject headings (MeSH) terms were incorporated where appropriate. The full research strategy and keywords are presented in Additional file 1: Table S1.

### Selection Process

Duplicated articles were removed using the Endnote software (version 20) [41]. Two authors performed the selection process independently, and disagreements between the two authors were solved by consensus. All articles were screened by the title and abstract. The full-text articles were screened for relevance using the eligibility criteria.

### Data Extraction

Microsoft Excel software was used to collect data from articles that met all the inclusion criteria. The data extraction process was performed by two authors independently to avoid any selection bias and data extraction flaws. The following data were extracted using a standardized spreadsheet and are presented in Table 2: Study identifiers, participants' information, study design, EIMD protocol, follow-up time points, outcomes and information about the supplementation protocol (supplement, leucine, isoleucine, and valine ratio, supplementation period, daily dosage, total dosage, placebo type).

**Table 2:** Summary of 18 studies included in this meta-analysis

Study	Participants	Study design	EIMD protocol	Follow-up time point	Outcomes	
Amirsasan et al. [46]	29 T. M. (23 ± 1)	DB Parallel L (n = 10) H (n = 10) P (n = 9)	Multi-joint and single-joint, Eccentric-dominant exercises (3 sets × 10 reps at 80% 1RM)	Pre, 24, 48 h	CK	NS.
					LDH	NS.
Areces et al. [68]	46 T. M + F. (41 ± 7)	DB Parallel B (n = 25) P (n = 21)	Marathon race (45 km)	Pre, ImPost	DOMS	NS.
Atashak and Baturak [69]	20 T. M. (22 ± 2)	SB Parallel B (n = 10) P (n = 10)	Eccentric-dominant exercise: 1 sets × 8 reps at 100% 1RM + 1 sets of 100% 1RM until volitional fatigue	Pre, ImPost, 24 h	CK	B < P 24 h
Barzegari [70]	40 T. M. (23 ± 4)	SB Parallel B (n = 20) P (n = 20)	Multi-joint and single-joint, Eccentric-dominant exercises: 4 sets × 10 reps at 80% 1RM	Pre, 24, 48 h	CK	NS.
					LDH	NS.
Dorrell and Gee [47]	5 T. M. (22 ± 1)	SB Crossover (5 days washout)	4 multi-joint barbells, Eccentric-dominant exercises: 4 sets × 8 reps at 75% 1RM	Pre, ImPost	DOMS	H + L < P
Gee and Deniel [63]	11 T. M. (25 ± 6)	SB Crossover (7 days washout)	4 multi-joint barbells, Eccentric-dominant exercises: 4 sets × 8 reps at 80% 1RM	Pre, 24 h	DOMS	NS.
Greer et al. [64]	9 U. M. (22 ± 3)	SB Crossover (8 days washout)	Cycling: 90 min at 55% VO^2^_max_	Pre, ImPost, 24, 48 h	CK	B < P 24, 48 h
					LDH	NS.
					DOMS	B < P 24 h
Howatson et al. [71]	12 T. M. (23 ± 2)	DB Parallel B (n = 6) P (n = 6)	Drop jump: 5 sets × 20 reps	Pre, 24, 48, 72, 96 h	CK	B < P 24 h
					DOMS	B < P 24, 48 h
Jackman et al. [72]	24 U. M. (NR.)	SB Parallel B (n = 12) P (n = 12)	Unilateral eccentric knee extension: 12 sets × 10 reps at 120% 1RM	Pre, ImPost, 24, 48, 72 h	DOMS	B < P 48, 72 h (flexion)
Kim et al. [73]	26 U. M. (22 ± 2)	DB Parallel B (n = 13) P (n = 13)	Cycling: 70% VO^2^_max_ until exhaustion	Pre, ImPost	CK	NS.
					LDH	NS.
Koba et al. [60]	16 T. M. (20 ± 1)	DB Parallel B (n = 8) P (n = 8)	Endurance exercise: 3 times/day (Total 40 km/day) for 5 days	Pre, ImPost	CK	NS.
					LDH	NS.
					DOMS	B < P
Koo et al. [65]	5 T. M. (17 ± 1)	SB Crossover (7 days washout)	Rowing race: 2000 m at the maximal intensity	Pre, ImPost,	CK	NS.
Ra et al. [61]	10 U. M. (22 ± 1)	DB Parallel B (n = 5) P (n = 5)	Eccentric-dominant exercise: 6 sets × 5 elbow flexions at 90% MVC	Pre, ImPost, 24, 48, 72 h, 96 h	CK	B < P 72, 96 h
					LDH	B < P 72, 96 h
					DOMS	B < P 72, 96 h
Sheikholeslami-Vatani and Ahmadi [66]	10 U. F. (22 ± 1.5)	DB Crossover (6 weeks washout)	Eccentric-dominant exercise: 5 sets × 12–15 reps at 50% 1RM	Pre, ImPost, 24 h	CK	NS.
					LDH	NS.
Shenoy et al. [74]	20 T. M. (20 ± 1.2)	DB Parallel B (n = 10) P (n = 10)	Drop jump: 5 sets × 20 reps	Pre, 24, 48 h	CK	B < P 24, 48 h
					DOMS	B < P 24, 48 h
Shimomura et al. [67]	12 U. F. (22 ± 1.6)	DB Crossover (11 weeks washout)	Eccentric-dominant Squat: 7 Sets × 20 reps with body weight	Pre, ImPost, 24, 48 h	CK	NS.
					DOMS	B < P 24, 48 h
VanDusseldorp et al. [62]	20 T. M. (22 ± 1.5)	DB Parallel B (n = 10) P (n = 10)	Eccentric:concentric, Squat: 10 sets × 8 reps at 70% 1RM + Split jump: 5 sets × 20 reps with body weight	Pre, ImPost, 24, 48, 72 h	CK	B < P 48 h
					DOMS	B < P 48, 72 h
Waldron et al. [75]	16 T. M + F. (22 ± 1.6; 22 ± 1)	DB Parallel B (n = 8) P (n = 8)	Eccentric-dominant Back squat:10 Sets × 6 reps at 70% 1RM	Pre, ImPost, 24, 48 h	CK	NS.
					DOMS	B < P 24, 48 h

M: Male; F: Female; T: Trained; U: Untrained; SB: Single blinded; DB: Double-blinded; B: BCAA group/condition; P: Placebo group/condition; L: Low dose group/condition; H: High dose group/condition; VO^2^_max_: maximal oxygen consumption; MVC: Maximal voluntary contraction; 1RM: One-repetition maximum; Pre: Before; ImPost: Immediately after; CK: Creatine kinase; LDH: lactate dehydrogenase; DOMS: Delayed onset of muscle soreness; NR: Not reported. NS: Not significant difference between BCAA and placebo

### Quality Assessment

Quality assessment in the selected studies was assessed with the randomized controlled trial (RCT) checklist from the Scottish Intercollegiate Guidelines Network (SIGN) [42]. The SIGN RCT checklist was developed to ensure a balance between methodological quality and practicality of use for authors and used in the present review because it is specific to the design of included studies. Two reviewers appraised each study based on these appraisal definitions, with any discrepancies resolved by an independent reviewer. A grade of ‘yes’, ‘no’, ‘can’t say’ or ‘not applicable’ was issued for each appraisal item. ‘Yes’ and ‘not applicable’ answers were indicative of a lower risk of bias; therefore, the total frequency of ‘yes’ and ‘not applicable’ answers were tallied to indicate overall methodological quality. The quality of each study was labelled as ‘high quality’, ‘acceptable’, ‘low quality’, or ‘unacceptable’.

### Statistical Analysis

Mean and standard deviation (SD) of the pre-and post-EIMD were extracted from the original studies. When data were not available, they were extracted from published figures using Web Plot Digitizer 4.6 (https://automeris.io/WebPlotDigitizer/, accessed on 20 September 2022) [43]. When the standard error of the mean (SEM) was reported in any study, SD was calculated as $SD=SEM \times \sqrt{n}$ where *n* equals group sample size. Mean difference and SD change (ΔSD) have been calculated and used to compute the effect sizes (ESs). According to the Cochrane Handbook for Systematic Reviews of Interventions [44], ΔSD was calculated using the following formula: $\mathrm{\Delta SD}= \sqrt{({SDpre}^{2}+ {SDpost}^{2}-2 \times Corr\left(pre,post\right)\times SDpre\times SDpost)}$.

The correlation coefficients were not reported in any included studies. Therefore, a correlation of 0.5 was assumed [45]. In the case of multi-arm studies (i.e., studies used two different doses of BCAA [46, 47]), the values of each dose were compared individually to the values of the placebo. Two studies was considered the minimum number of studies required to conduct a meta-analysis [44].

All meta-analyses was conducted using R programming language (version 4.2.1) [48, 49] with the Metafor package (version 3.8.1) [50]. ESs were calculated using the “escalc()” function. The random-effects model was conducted using the restricted maximum likelihood estimation to calculate model parameters and the inverse variance method was used to minimize the imprecision of the pooled effect estimate [44, 51]. The standardized mean difference (Hedges’ g) and 95% confidence intervals (CI) were calculated to investigate whether differential effects existed between BCAA and placebo interventions for each outcome measure at the same time point across all studies. Hedges’ g was used to measure ES and considered as small at g < 0.5, moderate at 0.5 < g < 0.8, and large at g > 0.8 [52]. Additionally, forest plots were used to illustrate point estimates of the ESs and 95% CIs. The between-study variance tau-square (Tau^2^), Q-test for heterogeneity, and *I*^*2*^ statistic assessed heterogeneity among studies, with I^2^ statistic considered as low at I^2^ < 50%, moderate at 50% < I^2^ < 75%, and high at I^2^ > 75% [53]. The prediction interval for the true outcomes was calculated [54]. Subgroup and meta-regression analyses were conducted respectively for categorical variables (i.e., study design [crossover vs parallel], blinding [single vs double], training status [trained vs untrained], and sex [male vs female vs both]) and continuous variables (i.e., daily dosage, total dosage, and supplementation period). Ten studies were considered the minimum number of studies from which to conduct a meta-regression analysis [44]. This analysis evaluated the sources of heterogeneity and how both categorical and continuous variables influenced measured outcomes across studies [44]. As indicated by Rothstein et al. [55], funnel plots’ potential asymmetries, Begg and Mazumdar’s rank correlation test [56], Egger’s linear regression test [57], and Duval and Tweedie’s trim and fill method [58] were conducted to identify publication bias. Studentized residuals and Cook’s distances were used to examine whether studies may be outliers and/or influential [59]. The stability of each study’s pooled ES was assessed via leave-one-out sensitivity analyses, involving the removal of individual studies from the analysis, and computing the excluded study’s impact on the overall effect estimate. The statistical significance level was set at *p* < 0.05 for all analyses.

## Results

### Study Selection for the Meta-analysis

**Fig. 1:**
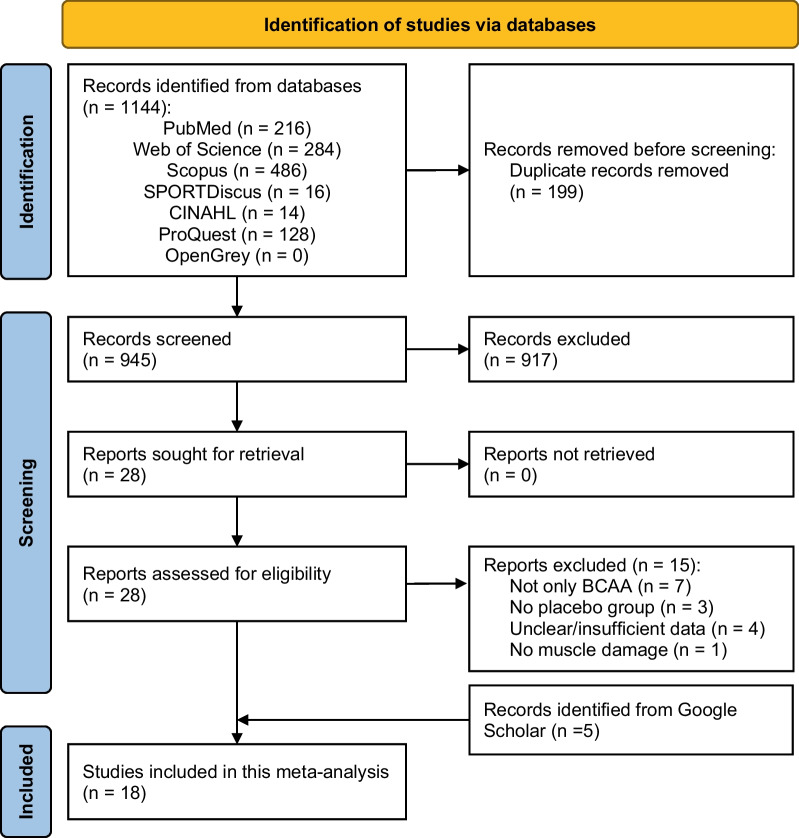
PRISMA flowchart of searching strategy and studies selection

The predefined search strategies identified a total of 1144 articles via online databases. A total of 199 duplicates were removed. After the screening, 945 articles remained based on the title and the abstract; of those 917 articles were excluded. After a careful review of 28 full-text articles, 13 articles were included. The additional search on Google scholar identified five articles, resulting in a total of 18 articles included in the quantitative analysis (i.e., meta-analysis) (Fig. 1).

### Study Characteristics

The characteristics of the 18 studies included in this meta-analysis are presented in Table 2. The studies were published between 2005 [60] and 2018 [61, 62]. The study design was a randomized controlled trial with a crossover design in six studies [47, 63–67] and a parallel design in 12 studies [46, 60–62, 68–75]. Moreover, participants were either single blinded in 7 studies [47, 63–65, 69, 70, 72] or double blinded in 11 studies [46, 60–62, 66–68, 71, 73–75].

A total of 331 participants were included across all studies, with 199 participants ingesting a BCAA supplement, and 200 participants ingesting placebo. Furthermore, 14 studies included only male participants, two studies included only female participants [66, 67], and two studies included both male and female participants [68, 75]. Additionally, participants were trained in 12 studies and untrained in 6 studies.

Out of the 18 included studies, participants performed resistance exercises in 13 studies and endurance exercises in five studies. For resistance exercises, the intensity varied from 50 to 120% of 1RM. Exercises were eccentric-dominant [46, 47, 61–63, 66, 69, 70, 72, 75], body weight-based [67], and drop jump sets [71, 74]. The intensity of cycling exercises varied from 55% [64] to 70% VO_2_ max [73]. Long-distance exercises [60, 68] and rowing races [65] were also used as endurance-damaging protocols.

Muscle damage and soreness were measured using CK, LDH, and DOMS. Studies measured only CK [65, 69], only DOMS [47, 63, 68, 72], CK and LDH [46, 66, 70, 73], CK and DOMS [62, 67, 71, 74, 75], or CK, LDH, and DOMS [60, 61, 64].

.BCAA supplementation strategies used in each study are presented in Table 3. BCAA doses varied from 3.15 to 29.3 g/day, or from 0.08 to 0.54 g/kg/day of body weight. The BCAA supplement was ingested for a period of from one to 28 days, with different employed strategies: only at the pre-load period [62, 65, 68, 74], only at the day of the damaging exercise (EIMD day) [47, 63, 64, 66, 67, 69, 70, 73], at pre-load and EIMD day [46, 61], at EIMD day and follow-up period [60, 72], or pre-load period, EIMD day, and follow-up period [71, 75].

**Table 3:** BCAA supplementation strategy of each study included in this meta-analysis

Study	LIV ratio	SP (day)				DD		TD (g)	Placebo
		PL	ED	FU	T	g/kg/day	g/day		
Amirsasan et al. [46]	02:01:01	6	1	–	7	0.21	15^a^	105	Dextrin
						0.45	32^a^	224	
Areces et al. [68]	02:01:01	7	–	–	7	NR.	5	35	Dextrose
Atashak and Baturak [69]	02:01:01	–	1	–	1	0.2	15^a^	15	Omega-3 Fatty acids
Barzegari [70]	02:01:01	–	1	–	1	0.45	34^a^	34	Dextrin
Dorrell and Gee [47]	02:01:01	–	1	–	1	NR.	6	6	Artificial sweetener
							18	18	
Gee and Deniel [63]	02:01:01	–	1	–	1	NR.	10	10	Apple and blackcurrant juice
Greer et al. [64]	2.5:1:1.5	–	1	–	1	NR.	5	5	Artificial sweetener
Howatson et al. [71]	02:01:01	7	1	4	12	NR.	20	240	Artificial sweetener
Jackman et al. [72]	2.1:1.2:1	–	1	2	3	NR.	29.3	87.9	Artificial sweetener
Kim et al. [73]	4.6:2:2.4	–	1	–	1	0.08	5^a^	5	Reverse osmosis water
Koba et al. [60]	02:01:01	–	5^**b**^	–	5	NR.	10	50	NR.
Koo et al. [65]	02:01:01	7	–	–	7	NR.	3.75	22	NR.
Ra et al. [61]	02:01:01	3	1	–	4	NR.	9.6	38.4	Starch
Sheikholeslami-Vatani and Ahmadi [66]	01:01:01	–	1	–	1	NR.	9	9	Dextrose
Shenoy et al. [74]	02:01:01	28	–	–	28	NR.	20	560	Artificial sweetener
Shimomura et al. [67]	2.3:1:1.2	–	1	–	1	NR.	5.5	5.5	Dextrin
VanDusseldorp et al. [62]	03:01:02	8	–	–	8	0.22	20^a^	160	Maltodextrin
Waldron et al. [75]	02:01:01	–	1	2	3	0.087	12^a^	36	Dextrose

LIV: Leucine, Isoleucine, and Valine ratio; SP: Supplementation period; PL: Pre-load; ED: EIMD day; FU: Follow-up; T: Total supplementation period; DD: Daily dosage; TD: Total Dosage; NR.: Not reported; ^a^Daily dosage (in g) was not stated in the article but estimated based on participants’ mean body mass; ^b^Supplementation during the training program for 5 days

### Effect of BCAA Supplementation on Creatine Kinase (CK)

**Fig. 2:**
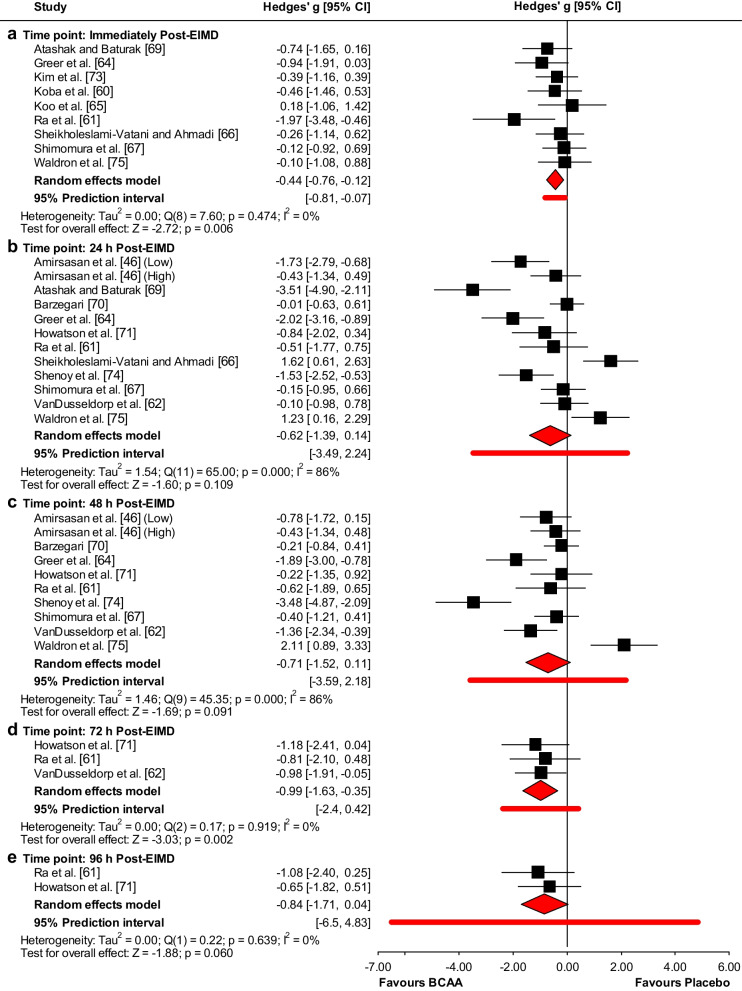
Forest plot of the effect of BCAA supplementation on CK levels compared to placebo at **a** immediately, **b** 24 h, **c** 48 h, **d** 72 h, and **e** 96 h post-EIMD

There was a positive effect of BCAA supplementation on CK levels immediately (ES = − 0.44 (low); 95% CI − 0.76 to − 0.12; *p* = 0.006) and 72 h post-EIMD (ES = − 0.99 (large); 95% CI − 1.63 to − 0.35; *p* = 0.002) (Fig. 2). However, no effects of BCAA supplementation were observed for CK levels at 24 h (ES = − 0.62 (moderate); 95% CI − 1.39 to 0.14; *p* = 0.109), 48 h (ES = − 0.71 (moderate); 95% CI − 1.52 to 0.11; *p* = 0.091), and 96 h post-EIMD (ES = − 0.84 (large); 95% CI − 1.71 to 0.04; *p* = 0.06) (Fig. 2). Heterogeneity was reported as significant at 24 h and 48 h (I^2^ = 86% (high)), but not immediately, 72 h, and 96 h post-EIMD (I^2^ = 0%) (Fig. 2).

Meta-regression analyses revealed a significant moderating effect of total dosage on CK levels at 48 h post-EIMD (Additional file 1: Fig. S1); for every 1 g increase, the ES decreased by 0.005 (95% CI − 0.01 to − 0.0004; *p* = 0.034). Additionally, a significant moderating effect of the supplementation period was reported at 48 h post-EIMD (Additional file 1: Fig. S2), indicating that for every 1-day increase, the ES decreased by 0.11 (95% CI − 0.2 to − 0.01; *p* = 0.025).

Subgroup analyses results are presented in Additional file 1: Table S3. Furthermore, study design (*p* = 0.02) and blinding (*p* = 0.04) immediately post-EIMD had a more significant impact on CK levels in parallel compared to crossover design studies and in single compared to double-blinded studies. Also, training status had a significant impact on CK levels (*p* = 0.02) at immediately post-EIMD in untrained compared to trained participants. Additionally, sex had a more significant impact on CK levels (*p* = 0.02 for immediately post, *p* = 0.002 for 24 h post, and *p* < 0.01 for 48 h post EIMD) in males compared to females and both males and females at all time points.

Funnel plots (Additional file 1: Fig. S3) showed no evidence of publication bias at any time point, which was confirmed by Begg and Mazumdar’s rank correlation test and Egger’s linear regression test (Additional file 1: Table S2). The Duval and Tweedie’s trim-and-fill analysis identified 2 studies to trim and a “true ES” of − 1.11 at 48 h post-EIMD, but no missing studies were identified immediately, 24 h, 72 h, and 96 h post-EIMD.

According to the studentized residuals and Cook’s distances, none of the studies were considered to be an outlier or overly influential at all time points.

Overall, the leave-one-out sensitivity analysis indicated that the effect of BCAA supplementation on CK is robust and not significantly driven by any single study immediately (Additional file 1: Fig. S4), 72 h (Additional file 1: Fig. S7), and 96 h post-EIMD (Additional file 1: Fig. S8). However, the leave-one-out sensitivity analyses demonstrated CK levels became significantly lower for the BCAA when we individually removed Waldron et al. [75] and Sheikholeslami-Vatani and Ahmadi [66] at 24 h (Additional file 1: Fig. S5) and Waldron et al. [75] at 48 h post-EIMD (Additional file 1: Fig. S6).

### Effect of BCAA Supplementation on Lactate Dehydrogenase (LDH)

**Fig. 3:**
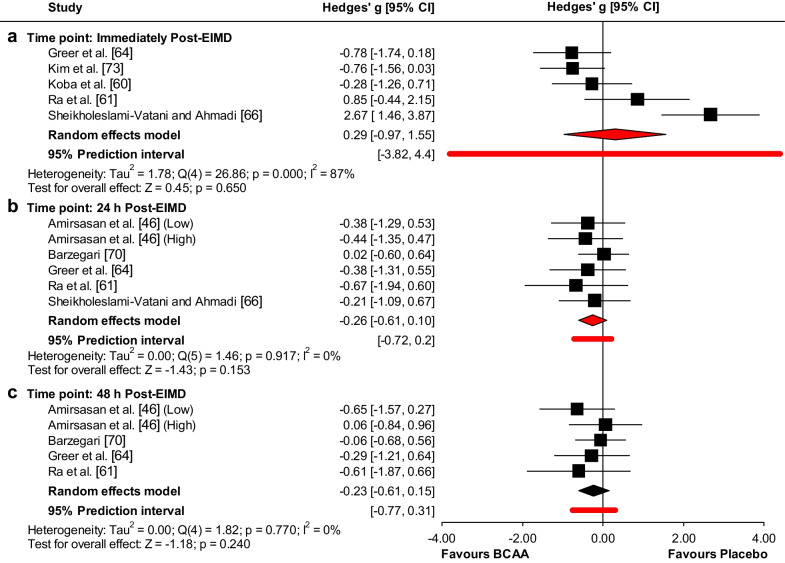
Forest plot of the effect of BCAA supplementation on LDH levels compared to placebo at **a** immediately, **b** 24 h, and **c** 48 h post-EIMD

No effects of BCAA supplementation on LDH levels were observed immediately (ES = 0.29 (low); 95% CI − 0.97 to 1.55; *p* = 0.65), 24 h (ES = − 0.26 (low); 95% CI − 0.61 to 0.1; *p* = 0.153), and 48 h post-EIMD (ES = − 0.23 (low); 95% CI − 0.61 to 0.15; *p* = 0.24) (Fig. 3). Heterogeneity was reported as significant at immediately post-EIMD (I^2^ = 87% (high)), but not at 24 h and 48 h post-EIMD (I^2^ = 0%) (Fig. 3). There were insufficient studies to conduct meta-regression analyses for any time point.

Funnel plot (Additional file 1: Fig. S9.B) showed evidence of publication bias at 24 h post-EIMD, which was confirmed by Egger’s linear regression test, but not by Begg and Mazumdar’s rank test (Additional file 1: Table S2). However, funnel plots (Additional file 1: Figs. S9.A and C) showed no evidence of publication bias immediately and 48 h post-EIMD, which was confirmed by Begg and Mazumdar’s rank correlation test and Egger’s linear regression test (Additional file 1: Table S2). The Duval and Tweedie’s trim-and-fill analysis identified 1 study at immediately post-EIMD to trim and a “true ES” of 0.57, 3 studies at 24 h to trim and a “true ES” of − 0.14, and 2 studies at 48 h post-EIMD to trim and a “true ES” of − 0.09.

The studentized residuals revealed Sheikholeslami-Vatani and Ahmadi [66] may be a potential outlier immediately post-EIMD. However, none of the studies could be considered to be an outlier at 24 h, and 48 h post-EIMD. According to Cook’s distances, Barzegari [70] could be considered to be overly influential at 24 h post-EIMD. None of the studies could be considered to be overly influential at immediately and 48 h post-EIMD.

Overall, the leave-one-out sensitivity analyses confirmed the reliability and stability of the current results of LDH levels (Additional file 1: Figs. S10-S12).

### Effect of BCAA Supplementation on Delayed Onset of Muscle Soreness (DOMS)

**Fig. 4:**
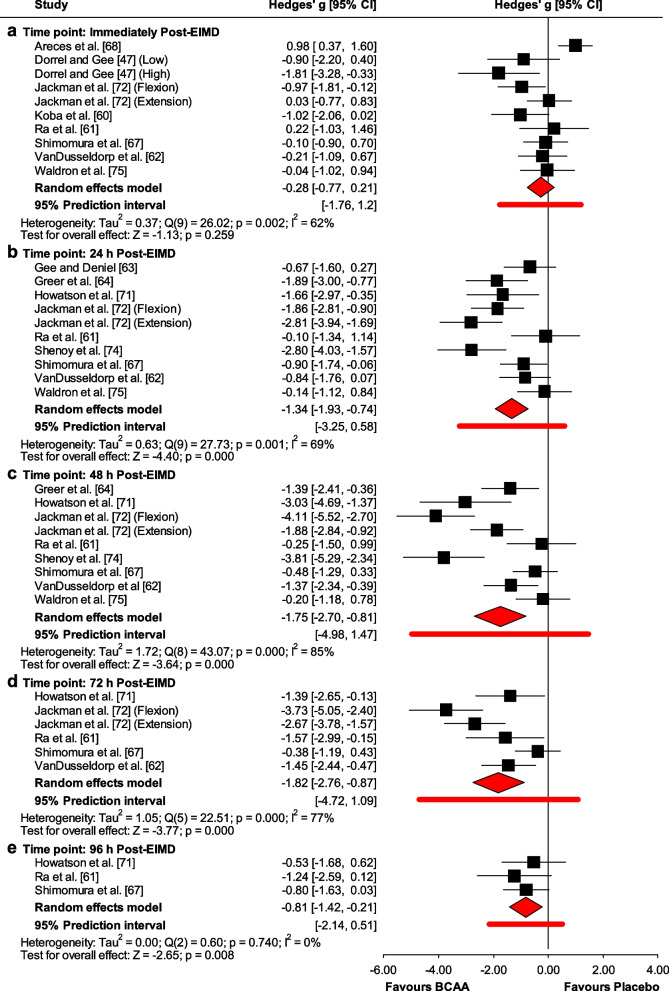
Forest plot of the effect of BCAA supplementation on DOMS compared to the placebo at **a** immediately, **b** 24 h, **c** 48 h, **d** 72 h, and **e** 96 h post-EIMD

No effect of BCAA supplementation on DOMS immediately post-EIMD (ES = − 0.28 (low); 95% CI − 0.77 to 0.21; *p* = 0.259) (Fig. 4). However, a positive effect of BCAA supplementation on DOMS was found at 24 h (ES = − 1.34 (large); 95% CI − 1.93 to − 0.74; *p* < 0.001), 48 h (ES = − 1.75 (large); 95% CI − 2.7 to − 0.81; *p* < 0.001), 72 h (ES = − 1.82 (large); 95% CI − 2.76 to − 0.87; *p* < 0.001), and 96 h post-EIMD (ES = − 0.82 (large); 95% CI − 1.42 to − 0.21; *p* = 0.008) (Fig. 4). Heterogeneity was significant at immediately, 24 h, 48 h and 72 h post-EIMD (I^2^ = 62% (moderate); I^2^ = 69% (moderate); I^2^ = 85% (high); I^2^ = 77% (high); respectively), but not at 96 h post-EIMD (I^2^ = 0%) (Fig. 4).

Meta-regression analyses revealed a significant moderating effect of daily dosage (Additional file 1: Fig. S13) at 24 h where for every 1 g increase, the ES decreased respectively by 0.06 (95% CI − 0.12 to − 0.003; *p* = 0.041).

Subgroup analyses results are presented in Additional file 1: Table S4. Moreover, the study design had a more significant impact on DOMS at 24 h and 48 h post-EIMD (*p* < 0.001 for both) for parallel compared to the crossover design. Also, blinding had a more significant effect on DOMS at 24 h (*p* < 0.001) and 48 h post-EIMD (*p* = 0.002) for single-blinded compared to double-blinded. The training status had a more significant impact on DOMS (*p* < 0.01 for 24 h and *p* = 0.01 for 48 h post EIMD) for untrained compared to trained participants at 24 h and for trained compared to untrained participants at 48 h post-EIMD. Sex had a more significant impact on DOMS at all time points (*p* = 0.035 for immediately and *p* < 0.001 for 24 h and 48 h post EIMD) for males compared to females, and both males and females.

A funnel plot (Additional file 1: Figs. S14.A and E) showed evidence of publication bias immediately and 96 h post-EIMD, which was confirmed by Egger’s linear regression test (Additional file 1: Table S2). Begg and Mazumdar’s rank correlation test showed no evidence of publication bias immediately post-EIMD (Additional file 1: Table S2). However, funnel plots (Additional file 1: Figs. S14.B-D) showed no evidence of publication bias at 24 h, 48, and 72 h post-EIMD, which was confirmed by Begg and Mazumdar’s rank correlation test and by Egger’s linear regression test (Additional file 1: Table S2). Duval and Tweedie’s trim-and-fill analysis identified one study to trim at immediately, 24 h, and 72 h post-EIMD and a “true ES” of − 0.18, − 1.09, and − 1.95, respectively, and two studies to trim at 96 h post-EIMD and a “true ES” of − 0.8. Contrarily, no missing studies were identified at 48 h post-EIMD.

According to the studentized residuals and Cook’s distances, none of the studies could be considered to be an outlier or overly influential at all time points.

Overall, the leave-one-out sensitivity analyses reported DOMS became significantly lower for BCAA when the study of Areces et al. [68] was individually removed at immediately post-EIMD (Additional file 1: Fig. S15). Otherwise, the leave-one-out sensitivity analysis indicated that the effect of BCAA supplementation on DOMS is not significantly driven by any single study from 24 to 96 h post-EIMD (Additional file 1: Fig. S16-S19).

### Quality Assessment

Results of the risk of bias analysis for each study are presented in Additional file 1: Table S5. Based on the agreed criteria of the SIGN RCT checklist, 13 studies were considered to be high quality [46, 47, 61–63, 66, 68–72, 74, 75] and four studies were considered to be of acceptable quality [60, 64, 65, 67, 73]. The concealment of the treatment groups from the research group was rarely completed. One study reported dropout during experimentation by four participants, creating dissimilarity between groups [73]. No study reported any conflict of interest.

## Discussion

The present meta-analysis revealed a positive effect of BCAA supplementation on recovery by decreasing CK levels at immediately and 72 h post-EIMD, but not at 24 h and 48 h post-EIMD. However, no effect of BCAA supplementation on LDH levels was observed at any time point. BCAA supplementation was beneficial by reducing DOMS from 24 to 96 h post-EIMD. Subgroup analyses identified significant moderating effects of study design, blinding, training status, and sex at immediately post-EIMD, and only sex at 24 h and 48 h post-EIMD on CK levels. Additionally, study design, blinding, and training status were identified as significant moderators at 24 h and 48 h post-EIMD on DOMS. Sex was a significant moderator for DOMS at all time points. Moreover, meta-regression analysis identified significant dose–response relationships for the total dosage and the supplementation period at 48 h on CK levels and only the daily dosage at 24 h on DOMS.

The present meta-analysis demonstrated BCAA supplementation had a positive impact on reducing CK levels at immediately post-EIMD and accelerating recovery at 72 h post-EIMD, but no effect was reported at 24 h and 48 h post-EIMD. Most of the selected studies were not in line with our findings at immediately, 24 h, and 48 h post-EIMD. None of the selected studies reported a significant impact of BCAA supplementation on CK levels at immediately post-EIMD. All the included studies reported a positive effect of BCAA on reducing CK levels; this might clarify our findings at immediately post-EIMD. Our results revealed no effect of BCAA on CK levels at 24 h and 48 h post-EIMD. However, beneficial effects of BCAA supplementation were observed immediately and 72 h post-EIMD. However, it is worth noting our results diverge from four other studies demonstrating significant effects of BCAA supplementation at different time points – specifically, 24 h [71], 48 h [62], and both 24 h and 48 h post-EIMD [64, 74]. While the timing of these observed effects differs from the current study, it is important to emphasize the outcomes of these four studies, despite their variance, still demonstrate positive influences of BCAA supplementation on muscle recovery and repair. This apparent discrepancy in timing highlights the complexity of the relationship between BCAA and post-exercise recovery, encouraging further exploration into the nuanced temporal aspects of their effects.

However, several studies presented a non-significant impact of BCAA on reducing CK levels, but with positive effects at 24 h and 48 h [46, 61, 67, 70]. Nevertheless, Waldron et al. [75] reported the BCAA group was significantly higher in CK levels than the placebo group at 24 h and 48 h post-EIMD, which was explained by the large SDs and random variations in CK levels through the following days due to the intra-individual CK levels [75]. ​With regard to the sensitivity analyses, the effect of BCAA became significant when Waldron et al. [75] was removed; this might explain the non-significant results at 24 h and 48 h post-EIMD. Lastly, the reduction of CK levels at 72 h in the BCAA group was supported by the selected studies [61, 62, 71, 74]. This finding may be explained by the long pre-load supplementation period in each study (i.e. seven [71], three [61], eight days [62], and 28 days [74]). Furthermore, our results were consistent with those reported by Khemtong et al. [29] at < 24 h, but not at 24 h and 48 h post-EIMD. It may be that the restricted inclusion criteria limited the number of included studies involving only trained male participants performing resistance exercises. Our results were in agreement with those reported in previous meta-analyses [28, 30], which reported significant effect of BCAA supplementation on CK levels reduction immediately post-EIMD [28] and at the following 24 h post-EIMD [30]. The present results disagree with previous meta-analyses at 24 h [29–31] and 48 h post-EIMD [29]. However, previous meta-analyses reported no effect on BCAA intake at 48 h post-EIMD [30, 31]. The contradictory results versus previous meta-analyses at 24 h and 48 h post-EIMD could be impacted by sex; indeed, our subgroup analyses indicated those studies involving only male participants had a significant impact on CK levels. Furthermore, BCAA had a non-significant impact on CK levels at 72 h post-EIMD [31]. The partial disparity with previous meta-analyses might be related to the number of studies included, with more studies analysed allowing for greater statistical power [76].

Our results suggest BCAA supplementation had no impact on LDH levels at all time points post-EIMD. As mentioned above, LDH is an enzyme that assists the production of lactate from pyruvate, and is a marker for contractile element damage in muscle [24] which occurs during the early phases of inflammation [77]. Consistently with the included studies [46, 60, 61, 64, 70, 73], BCAA supplementation had no effect on LDH levels reduction. Regarding delayed effects of supplementation, Ra et al. [61] reported BCAA significantly decreased LDH levels at 72 h and 96 h post-EIMD. The beneficial impact of BCAA supplementation may be related to trials with a long supplementation period. In addition, LDH level increases may be affected by exercise conditions, the primary site of muscle damage, and training status [30]. The differing responses of LDH and CK in our study likely stem from methodological factors. Factors such as assay sensitivity, sampling timing, and participant characteristics could affect measurement reliability. Moreover, the specific muscle groups targeted by the exercise protocol could shape enzyme release patterns [78]. Focusing more deeply on these methodological facets could aid in clarifying the observed differences in LDH and CK response. Existing literature suggests LDH might be less specific to muscle damage than CK due to its presence in various tissues, including red blood cells and the liver, potentially introducing background noise, and explaining the subdued LDH responses compared to CK in our study [79]. The effects of BCAA on CK efflux could arise from their potential influence on muscle repair, regeneration, protein synthesis, immune modulation, and energy metabolism [71]. This distinct role of BCAA warrants further investigation into their mechanistic interactions with CK efflux pathways. Our results are in accordance with previous meta-analyses regarding no reduction in LDH levels with BCAA supplementation [28–31].

Our findings revealed BCAA supplementation had a significant impact on lowering DOMS from 24 to 96 h, but not immediately post-EIMD. Similarly, BCAA supplementation had no effect on DOMS reduction immediately post-EIMD in most of the selected studies. It should be acknowledged that only Koba et al. [60] investigated the effect of BCAA intake over five days of an intensive endurance training program with three training sessions per day. This reduction of DOMS levels in the BCAA group can be explained by the daily intake of BCAA during the training program days, which may affect DOMS at the end of the training program [55]. Consistent with our findings, BCAA supplementation had a beneficial impact on reducing DOMS post-EIMD at 24 h only [64], at 24 h and 48 h [67, 71, 74, 75], and at 48 h and 72 h post-EIMD [62, 72]. In addition, Ra et al. [61] revealed a significant effect of BCAA on DOMS at 72 h and 96 h. This result was explained by the increase in β-hydroxy β-methyl butyric (3HMB) levels during exercise, which may have been linked to the beneficial effects of BCAA intake on DOMS [61]. The mechanism producing muscle soreness is not fully understood, although some studies have suggested that oxidative stress and exercise-induced free radicals, as well as inflammation in connective tissue components, may be involved [80, 81], potentially sensitizing nociceptors [82]. BCAA supplementation may reduce oxidative stress and free radical levels in athletes [83]. Furthermore, Jackman et al. [72] suggested the increase in food intake, specifically amino acids, could potentially be linked to reductions in soreness. However, it is important to note this hypothesis lacks supporting evidence or a proposed mechanism, making it difficult to either endorse or refute the theory. Nevertheless, it is possible that the uptake of BCAA for protein synthesis may contribute to a decrease in the secondary damage phase. This, in turn, could limit the overall extent of damage, leading to a reduction in the occurrence of soreness [71]. Previous research suggests the reason for BCAAs' ability to decrease CK release and minimize muscle damage may be attributed to their improved availability of nitrogen and ability to uphold membrane integrity during the secondary phase of muscle damage following eccentric exercise [71, 74]. It is worth noting that the effect of glutamine may be a possible explanation for the influence of BCAA on muscle soreness. Glutamine is a free amino acid prevalent in plasma and skeletal muscle and is involved in protein synthesis [84]. In general, glutamine is significantly used by inflammatory and damaged cells to reduce the severity of damage and pain. BCAA can also be transaminated to glutamine in order to increase glutamine synthesis [17, 85]. Thus, the mechanisms of BCAA effects on muscle soreness are currently unclear. The results of the current study were consistent with a previous meta-analysis at 24 h and 48 h [27], revealing a significant impact of BCAA on DOMS. Additionally, our results were in line with Rahimlou et al. [31] at all time except 96 h post-EIMD. A recent meta-analysis reported BCAA intake may reduce DOMS at 24 h and 72 h [32], but not at 48 h and 96 h post-EIMD, which partly aligned with our results. Previous meta-analyses [29, 30] revealed no effect of BCAA on DOMS at all time points.

With respect to our meta-regression analyses, the supplementation period significantly predicted CK levels at 48 h post-EIMD. The total dosage presented the interaction effect between the daily dosage and the supplementation period, which was a significant predictor of CK levels at 48 h post-EIMD. BCAA supplementation with a high dosage during a short period may have no positive effects, while a long supplementation period could elicit positive effects on lowering CK levels immediately post-EIMD, as suggested in the present study. Additionally, long supplementation periods (> 1 day) may be better than short periods (1 day) [28] in lowering CK levels. Studies using a longer supplementation period recorded a high total dosage of BCAA and high ESs for CK levels [46, 62, 71, 74] and DOMS [71, 72, 74] at 48 h post-EIMD. Moreover, two studies [46, 47] investigated and compared two different dosages resulting in contradictory findings, i.e. higher ES for low dose (210 mg/kg) compared to high dose (450 mg/kg) in reducing muscle damage biomarkers at 24 h and 48 h post-EIMD [46]. As previously mentioned, ingesting higher doses during a shorter period of time appears to have limited to no effects on EIMD [20]. Consistently, 18 g of BCAA is not effective for lowering muscle soreness compared to 6 g at immediately post-EIMD [47]. Furthermore, the optimal daily dosage of BCAA is not yet established. Furthermore, according to Fouré and Bendahan [20], supplementation with 200 mg/kg/day of BCAA for 4 to 10 days does not seem long enough to provide favourable effects [20].

Regarding subgroup analyses results, the study design and blinding influenced CK levels immediately and DOMS scores at 24 h and 48 h post-EIMD, which showed a higher effect for parallel design compared to crossover design and a higher effect for single-blinded studies compared to double-blinded studies. This finding is contradicted by Doma et al. [27], with the explanation that a crossover design is used in order to minimize inter-individual variability [27]. Thus, due to differences in results, the findings do not allow for a reasonable consensus on the optimal study design for studies assessing the effect of BCAA on the recovery process of muscle damage biomarkers and muscle soreness. Additionally, training status had a greater impact on CK levels for untrained participants immediately and on DOMS for trained participants at 24 h and 48 h post-EIMD. There was an unclear link between training status and the effectiveness of BCAA supplementation on CK levels and DOMS reduction. Indeed, some effects of BCAA supplementation in trained participants may be linked to better muscle adaptation, such as improved mobilization and activation of anti-inflammatory cells (i.e., T regulatory cells (Tregs)) [86, 87], where BCAA supplementation increases Treg cell regeneration and activation [87]. Likewise, sex effect was reported for males on reducing CK levels and DOMS. Studies involving female participants mostly reported low positive ESs, even negative effects [66, 67, 75], which could be explained by changes in female hormones during menstruation, as oestrogen has been shown to affect the exercise-induced response in plasma muscle damage indicators [88, 89]. It should be acknowledged that the results of subgroup and meta-regression analyses should be interpreted with caution due to their observational nature [90].

To the authors’ current knowledge, this is the first meta-analysis examining the effect of BCAA supplementation at different follow-up time points, from immediately post-EIMD to 48 h, 72 h, and 96 h post-EIMD for LDH, CK, and DOMS, respectively. The strengths of the current study include a comprehensive review of the previous studies and a careful assessment of their methodological quality. Additionally, no language or year limitations were set on the search processing. Furthermore, this meta-analysis is limited to studies using only BCAA, with no co-ingestion of other essential amino acids or other ingredients with BCAA in order to avoid confounding effects. Moreover, included studies used different study designs, and involved trained and untrained male and female participants, with different BCAA dosages and supplementation periods. These factors were used to perform subgroup and meta-regression analyses to identify the source of diversity in CK and DOMS results. However, the number of studies evaluating the effects of BCAA supplementation on CK levels and DOMS, respectively at 72 h and 96 h post-EIMD, is small, limiting the ability to conduct subgroup and meta-regression analyses. Additionally, the small number of studies evaluating the effects of BCAA supplementation on LDH levels and DOMS limited the subgroup and meta-regression analyses [44]. The absence of reported menstrual cycle or hormonal contraceptive status of female participants in previous studies is a significant limitation, considering the demonstrated influence of estrogen and progesterone profiles on EIMD and DOMS throughout the menstrual cycle (Romero-Parra et al. [89]). Additionally, Smith-Ryan et al. [91] found circulating CK levels are elevated during menstruation, further emphasizing the importance of considering hormonal variations when investigating exercise-related outcomes in women's health. Future studies exploring the interplay between menstrual cycle phases, hormonal fluctuations, and exercise-induced muscle responses should also consider the potential impact of BCAA supplementation on females' physiology during different menstrual phases. The lack of information regarding BCAA supplementation and daily dietary intake in the majority of the studies included prevented an assessment of whether the participants met their daily EAA/BCAA requirements. Therefore, the efficacy of BCAA supplementation in providing therapeutic/protective effects on EIMD comes into question, as it may not solely serve as a means to fulfil their daily amino acid requirements. Only a few individual studies [62, 66] examined the daily intake of BCAA during the supplementation protocol. Future studies should control and mention the overall daily protein, especially BCAA, intake during the supplementation period. The findings of this study suggest longer supplementation periods may provide positive effects of BCAA. Future studies should investigate the long-term effects of BCAA and examine the related mechanisms. Another limitation of the inter-study comparison was the variety of exercise protocols utilized. Studies using either resistance or endurance protocols differ in intensity, volume, and muscles included in exercises, which may lead to a diversity of outcomes. More research is needed to fully understand the effects of BCAA on the recovery process (i.e., muscle damage biomarkers and muscle soreness). It is well known CK levels are a marker of muscle damage resulting from strenuous workouts. BCAA may help with CK levels post exercise, thus benefiting athletes’ recovery. However, the low number of current studies limits the possibility of in-depth investigation. Further research is needed to ascertain if BCAA enhance recovery amidst higher CK levels, benefiting intense exercise recovery. Controlled studies across varied exercise intensities and damage levels could clarify the CK-BCAA-muscle recovery relationship. The widely used 2:2:1 leucine, isoleucine, and valine ratio was prevalent in our analysis. Future studies should explore how BCAA supplement quality influences the leucine, isoleucine, and valine ratio's impact and how varying ratios might affect muscle-related outcomes. Furthermore, future studies should *(i)* measure biomarkers and muscle soreness for a prolonged follow-up period up to 72 h or 96 h post-EIMD; *(ii)* compare different dosages of BCAA; *(iii)* examine the effect of BCAA supplementation timing (i.e., pre-, post-, or pre-and post-EIMD); *(iv)* explore the effects of BCAA with different leucine, isoleucine, and valine ratios; *(v)* evaluate the impact of BCAA intake on oxidative stress responses; *(vi)* and assess the co-ingestion effect of BCAA with another amino acid (e.g., taurine), other ergogenic aids (e.g., creatine), or other ingredients.

## Conclusion

BCAA supplementation appears to reduce CK levels within the first 24 h. and at 72 h post-EIMD and reduce DOMS from 24 h up to 96 h post-EIMD but has no beneficial effect on LDH levels. Furthermore, BCAA supplementation may be used as an effective strategy to accelerate the recovery process after intense exercise. As a major observation, the dose–response relationship for multiple factors was evident, suggesting ingestion of either low or high dosage across a longer supplementation period (thus resulting in higher total dosage) may increase BCAA efficacy when ingested after EIMD.

### Supplementary Information

**Additional file 1: Table S1.** Terms combinations and search results on each database.** Table S2**. Results of the Begg and Mazumdar’s Rank Correlation Test and Egger’s Linear Regression Test.** Table S3.** Subgroup analyses of categorical variables on CK levels at Immediately, 24 h, and 48 h post-EIMD.** Table S4**. Subgroup analyses of categorical variables on DOMS at Immediately, 24 h, and 48 h post-EIMD.** Table S5**. The SIGN RCT checklist for each included study.** Fig. S1.** Regression of hedges’ g on total dosage of BCAA of CK at 48 h post-EIMD.** Fig. S2.** Regression of Hedges’ g on supplementation period of CK at 48 h post-EIMD.** Fig. S3.** Funnel plots of CK levels at** A** immediately,** B** 24 h,** C** 48 h,** D** 72 h, and** E** 96 h post-EIMD.** Fig. S4.** Sensitivity analysis showing reliability and stability of CK levels at immediately post-EIMD.** Fig. S5.** Sensitivity analysis showing reliability and stability of CK levels at 24 h post-EIMD.** Fig. S6.** Sensitivity analysis showing reliability and stability of CK levels at 48 h post-EIMD.** Fig. S7.** Sensitivity analysis showing reliability and stability of CK levels at 72 h post-EIMD.** Fig. S8.** Sensitivity analysis showing reliability and stability of CK levels at 72 h post-EIMD.** Fig. S9.** Funnel plots of LDH levels at** A** immediately,** B** 24 h, and** C** 48 h post-EIMD.** Fig. S10.** Sensitivity analysis showing reliability and stability of LDH levels at immediately post-EIMD.** Fig. S11.** Sensitivity analysis showing reliability and stability of LDH levels at 24 h post-EIMD.** Fig. S12.** Sensitivity analysis showing reliability and stability of LDH levels at 48 h post-EIMD.** Fig. S13.** Regression of Hedges’ g on daily dosage of DOMS at 24 h post-EIMD.** Fig. S14.** Funnel plots of DOMS at** A** immediately,** B** 24 h,** C** 48 h,** D** 72 h, and** E** 96 h post-EIMD.** Fig. S15.** Sensitivity analysis showing reliability and stability of DOMS levels at immediately post-EIMD.** Fig. S16.** Sensitivity analysis showing reliability and stability of DOMS levels at 24 h post-EIMD.** Fig. S17**. Sensitivity analysis showing reliability and stability of DOMS levels at 48 h post-EIMD.** Fig. S18**. Sensitivity analysis showing reliability and stability of DOMS levels at 72 h post-EIMD.** Fig. S19.** Sensitivity analysis showing reliability and stability of DOMS levels at 96 h post-EIMD. PRISMA checklist.

## Data Availability

Not applicable.

## Abbreviations

3HMB: β-Hydroxy β-methyl butyric
BCAA: Branched-chain amino acids
CI: Confidence interval
CK: Creatine kinase
DOMS: Delayed onset of muscle soreness
EAAs: Essential amino acids
EIMD: Exercise-included muscle damage
ES: Effect size
fTRP: Free tryptophan
LDH: Lactate dehydrogenase
PRISMA: The preferred reporting items for systematic reviews and meta-analysis
SD: Standard deviation
TCA: Tricarboxylic acid cycle
Treg: T regulatory cells
