# Supplementary material for: Attenuating Muscle Damage Biomarkers and Muscle Soreness After an Exercise-Induced Muscle Damage with Branched-Chain Amino Acid (BCAA) Supplementation: A Systematic Review and Meta-analysis with Meta-regression
Source: Sports Med Open. 2024 Apr 16;10:42. doi: 10.1186/s40798-024-00686-9 (PMC11021390; doi:10.1186/s40798-024-00686-9)
Supplement: Supplementary file 1 — Additional file 1: Table S1. Terms combinations and search results on each database. Table S2. Results of the Begg and Mazumdar’s Rank Correlation Test and Egger’s Linear Regression Test. Table S3. Subgroup analyses of categorical variables on CK levels at Immediately, 24 h, and 48 h post-EIMD. Table S4. Subgroup analyses of categorical variables on DOMS at Immediately, 24 h, and 48 h post-EIMD. Table S5. The SIGN RCT checklist for each included study. Fig. S1. Regression of hedges’ g on total dosage of BCAA of CK at 48 h post-EIMD. Fig. S2. Regression of Hedges’ g on supplementation period of CK at 48 h post-EIMD. Fig. S3. Funnel plots of CK levels at A immediately, B 24 h, C 48 h, D 72 h, and E 96 h post-EIMD. Fig. S4. Sensitivity analysis showing reliability and stability of CK levels at immediately post-EIMD. Fig. S5. Sensitivity analysis showing reliability and stability of CK levels at 24 h post-EIMD. Fig. S6. Sensitivity analysis showing reliability and stability of CK levels at 48 h post-EIMD. Fig. S7. Sensitivity analysis showing reliability and stability of CK levels at 72 h post-EIMD. Fig. S8. Sensitivity analysis showing reliability and stability of CK levels at 72 h post-EIMD. Fig. S9. Funnel plots of LDH levels at A immediately, B 24 h, and C 48 h post-EIMD. Fig. S10. Sensitivity analysis showing reliability and stability of LDH levels at immediately post-EIMD. Fig. S11. Sensitivity analysis showing reliability and stability of LDH levels at 24 h post-EIMD. Fig. S12. Sensitivity analysis showing reliability and stability of LDH levels at 48 h post-EIMD. Fig. S13. Regression of Hedges’ g on daily dosage of DOMS at 24 h post-EIMD. Fig. S14. Funnel plots of DOMS at A immediately, B 24 h, C 48 h, D 72 h, and E 96 h post-EIMD. Fig. S15. Sensitivity analysis showing reliability and stability of DOMS levels at immediately post-EIMD. Fig. S16. Sensitivity analysis showing reliability and stability of DOMS levels at 24 h post-EIMD. Fig. S17. Sensitivity analysi [file 40798_2024_686_MOESM1_ESM.docx]

**Attenuating Muscle Damage Biomarkers and Muscle Soreness After an Exercise-Induced Muscle Damage with Branched-Chain Amino Acid (BCAA) Supplementation: A Systematic review and Meta-Analysis with Meta-regression**

**Table S1** Terms combinations and search results on each database

| **Database** | **Keywords** |
| --- | --- |
| **PubMed**  **(September 13^th^, 2022)** | **(**"Amino Acids, Branched-Chain"[Mesh] OR "Branched chain amino acid*"[tw] OR "Branched chain aminoacid*"[tw] OR BCAA*[tw]**)**  **AND (**"Exercise"[Mesh] OR Exercise[tw] OR EIMD[tw] OR Training[tw] OR "Physical activit*"[tw] OR "Physical exercise*"[tw] OR "Acute exercise*"[tw] OR "Aerobic exercise*"[tw] OR "Anaerobic exercise*"[tw] OR "Isometric exercise*"[tw] OR "Resistance training"[tw] OR "Endurance training"[tw]**)**  **AND (**"Creatine Kinase"[Mesh] OR "L-Lactate Dehydrogenase"[Mesh] OR "Visual Analog Scale"[Mesh] OR "Muscle damage "[tw] OR recovery[tw] OR "Muscle performance "[tw] OR "Physical performance*"[tw] OR "Muscle recovery"[tw] OR "Physical performance recovery"[tw] OR "Creatine kinase"[tw] OR "Creatine phosphokinase"[tw] OR CK[tw] OR CPK[tw] OR "Lactate dehydrogenase"[tw] OR LDH[tw] OR Myoglobin[tw] OR “aspartate aminotransferase”[tw] OR ASAT[tw] OR “aspartate transaminase”[tw] OR AST[tw] OR “alanine aminotransferase”[tw] OR ALAT[tw] OR “alanine transaminase”[tw] OR ALT[tw] OR "Visual analogue scale"[tw] OR VAS[tw] OR "Delayed onset of muscle soreness"[tw] OR DOMS[tw] OR "Muscle soreness"[tw]**)**  **NOT (**rat[tw] OR patient*[tw] OR desease*[tw] OR sedentary*[tw] OR animal[tw] OR inacti*[tw] OR Mice[tw] OR horse[tw]**)**  **216 results** |
| **Web of Science**  **(September 13^th^, 2022)** | **(**Branched chain amino acid OR Branched chain aminoacid OR BCAA**)**  **AND (**Exercise OR EIMD OR Exercise included muscle damage OR Training OR Physical activity OR Physical exercise OR Acute exercise OR Aerobic exercise OR Anaerobic exercise OR Isometric exercise OR Resistance training OR Endurance training**)**  **AND (**Creatine kinase OR Creatine phosphokinase OR CK OR CPK OR Lactate dehydrogenase OR LDH OR Myoglobin OR aspartate aminotransferase OR ASAT OR aspartate transaminase OR AST OR alanine aminotransferase OR ALAT OR alanine transaminase OR ALT OR Visual analogue scale OR VAS OR Delayed onset of muscle soreness OR DOMS OR Muscle soreness OR Muscle damage OR recovery OR Muscle performance OR Physical performance OR Muscle recovery OR Physical performance recovery**)**  **NOT (**rats OR patient OR desease OR sedentary OR animal OR inactive OR mice OR horse**)**  **284 results** |
| **Scopus**  **(September 13^th^, 2022)** | **(**“Branched chain amino acid” OR “Branched chain aminoacid” OR BCAA**)**  **AND (**Exercise OR EIMD OR “Exercise included muscle damage” OR Training OR “Physical activity” OR “Physical exercise” OR “Acute exercise” OR “Aerobic exercise” OR “Anaerobic exercise” OR “Isometric exercise” OR “Resistance training” OR “Endurance training”**)**  **AND** “Creatine kinase” OR “Creatine phosphokinase” OR CK OR CPK OR “Lactate dehydrogenase” OR LDH OR Myoglobin OR “aspartate aminotransferase” OR ASAT OR “aspartate transaminase” OR AST OR “alanine aminotransferase” OR ALAT OR “alanine transaminase” OR ALT OR “Visual analogue scale” OR VAS OR “Delayed onset of muscle soreness” OR DOMS OR “Muscle soreness” OR “Muscle damage” OR recovery OR “Muscle performance” OR “Physical performance” OR “Muscle recovery” OR “Physical performance recovery”**)**  **NOT (**rat OR rats OR patient OR desease OR sedentar OR animal OR inacti OR mice OR horse**)**  **486 results** |
| **SPORTDiscus**  **(September 13^th^, 2022)** | **(**“Branched chain amino acid” OR “Branched chain aminoacid” OR BCAA**)**  **AND (**Exercise OR EIMD OR “Exercise included muscle damage” OR Training OR “Physical activity” OR “Physical exercise” OR “Acute exercise” OR “Aerobic exercise” OR “Anaerobic exercise” OR “Isometric exercise” OR “Resistance training” OR “Endurance training”**)**  **AND** “Creatine kinase” OR “Creatine phosphokinase” OR CK OR CPK OR “Lactate dehydrogenase” OR LDH OR Myoglobin OR “aspartate aminotransferase” OR ASAT OR “aspartate transaminase” OR AST OR “alanine aminotransferase” OR ALAT OR “alanine transaminase” OR ALT OR “Visual analogue scale” OR VAS OR “Delayed onset of muscle soreness” OR DOMS OR “Muscle soreness” OR “Muscle damage” OR recovery OR “Muscle performance” OR “Physical performance” OR “Muscle recovery” OR “Physical performance recovery”**)**  **NOT (**rat OR rats OR patient OR desease OR sedentar OR animal OR inacti OR mice OR horse**)**  **16 results** |
| **CINAHL**  **(September 13^th^, 2022)** | **(**“Branched chain amino acid” OR “Branched chain aminoacid” OR BCAA**)**  **AND (**Exercise OR EIMD OR “Exercise included muscle damage” OR Training OR “Physical activity” OR “Physical exercise” OR “Acute exercise” OR “Aerobic exercise” OR “Anaerobic exercise” OR “Isometric exercise” OR “Resistance training” OR “Endurance training”**)**  **AND** “Creatine kinase” OR “Creatine phosphokinase” OR CK OR CPK OR “Lactate dehydrogenase” OR LDH OR Myoglobin OR “aspartate aminotransferase” OR ASAT OR “aspartate transaminase” OR AST OR “alanine aminotransferase” OR ALAT OR “alanine transaminase” OR ALT OR “Visual analogue scale” OR VAS OR “Delayed onset of muscle soreness” OR DOMS OR “Muscle soreness” OR “Muscle damage” OR recovery OR “Muscle performance” OR “Physical performance” OR “Muscle recovery” OR “Physical performance recovery”**)**  **NOT (**rat OR rats OR patient OR desease OR sedentar OR animal OR inacti OR mice OR horse**)**  **14 results** |
| **ProQuest**  **(September 13^th^, 2022)** | **(**“Branched chain amino acid” OR “Branched chain aminoacid” OR BCAA**)**  **AND (**Exercise OR EIMD OR “Exercise included muscle damage” OR Training OR “Physical activity” OR “Physical exercise” OR “Acute exercise” OR “Aerobic exercise” OR “Anaerobic exercise” OR “Isometric exercise” OR “Resistance training” OR “Endurance training”**)**  **AND** “Creatine kinase” OR “Creatine phosphokinase” OR CK OR CPK OR “Lactate dehydrogenase” OR LDH OR Myoglobin OR “aspartate aminotransferase” OR ASAT OR “aspartate transaminase” OR AST OR “alanine aminotransferase” OR ALAT OR “alanine transaminase” OR ALT OR “Visual analogue scale” OR VAS OR “Delayed onset of muscle soreness” OR DOMS OR “Muscle soreness” OR “Muscle damage” OR recovery OR “Muscle performance” OR “Physical performance” OR “Muscle recovery” OR “Physical performance recovery”**)**  **NOT (**rat OR rats OR patient OR desease OR sedentar OR animal OR inacti OR mice OR horse**)**  **128 results** |
| **OpenGray**  **(September 13^th^, 2022)** | **(**Branched chain amino acid OR Branched chain aminoacid OR BCAA**)**  **AND (**Exercise OR EIMD OR Exercise included muscle damage OR Training OR Physical activity OR Physical exercise OR Acute exercise OR Aerobic exercise OR Anaerobic exercise OR Isometric exercise OR Resistance training OR Endurance training**)**  **AND (**Creatine kinase OR Creatine phosphokinase OR CK OR CPK OR Lactate dehydrogenase OR LDH OR Myoglobin OR aspartate aminotransferase OR ASAT OR aspartate transaminase OR AST OR alanine aminotransferase OR ALAT OR alanine transaminase OR ALT OR Visual analogue scale OR VAS OR Delayed onset of muscle soreness OR DOMS OR Muscle soreness OR Muscle damage OR recovery OR Muscle performance OR Physical performance OR Muscle recovery OR Physical performance recovery**)**  **NOT (**rats OR patient OR desease OR sedentary OR animal OR inactive OR mice OR horse**)**  **0 results** |

**Table S2** Results of the Begg and Mazumdar’s Rank Correlation Test and Egger’s Linear Regression Test

|  |  | **Begg and Mazumdar’s Rank Correlation Test** | | **Egger’s Linear Regression Test** | | |
| --- | --- | --- | --- | --- | --- | --- |
| **Outcomes** | **Time point** | Kendall’s tau | p | t | df | p |
| **CK** | Immediately post-EIMD | -0.167 | 0.612 | -1.387 | 7 | 0.208 |
|  | 24 h post-EIMD | -0.424 | 0.063 | -1.526 | 10 | 0.158 |
|  | 48 h post-EIMD | -0.333 | 0.216 | -0.76 | 8 | 0.469 |
|  | 72 h post-EIMD | 0.333 | 1 | 0.041 | 1 | 0.974 |
|  | 96 h post-EIMD | - | - | - | - | - |
| **LDH** | Immediately post-EIMD | 0.8 | 0.083 | 2.3 | 3 | 0.105 |
|  | 24 h post-EIMD | -0.733 | 0.056 | -6.705 | 4 | 0.003 |
|  | 48 h post-EIMD | -0.4 | 0.483 | -1.405 | 3 | 0.255 |
| **DOMS** | Immediately post-EIMD | -0.422 | 0.108 | -2.997 | 8 | 0.017 |
|  | 24 h post-EIMD | -0.111 | 0.728 | -0.751 | 8 | 0.474 |
|  | 48 h post-EIMD | -0.279 | 0.359 | -2.086 | 7 | 0.075 |
|  | 72 h post-EIMD | -0.467 | 0.272 | -1.495 | 4 | 0.209 |
|  | 96 h post-EIMD | 1 | 0.333 | -58.513 | 1 | 0.011 |

**Table S3** Subgroup analyses of categorical variables on CK levels at Immediately, 24 h, and 48 h post-EIMD

| **Follow-up time point**  Subgroup | | **ES** | **95% CI** | **p** | **Test of Moderators** |
| --- | --- | --- | --- | --- | --- |
| **Study design** | **Immediately post-EIMD** | | | | |
|  | Crossover | -0.41 | -0.98 to 0.17 | 0.17 | Q = 7.43, p = 0.02 |
|  | Parallel | -0.46 | -0.84 to -0.08 | 0.02 |  |
|  | **24 h post-EIMD** | | | | |
|  | Crossover | -0.17 | -2.2 to 1.86 | 0.87 | Q = 3.44, p = 0.18 |
|  | Parallel | -0.77 | -1.6 to 0.05 | 0.07 |  |
|  | **48 h post-EIMD** | | | | |
|  | Crossover | -1.09 | -2.55 to 0.36 | 0.14 | Q = 3.56, p = 0.17 |
|  | Parallel | -0.6 | -1.61 to 0.4 | 0.24 |  |
| **Blinding** | **Immediately post-EIMD** | | | | |
|  | Single | -0.61 | -1.19 to -0.2 | 0.04 | Q = 7.86, p = 0.04 |
|  | Double | -0.37 | -0.75 to 0.01 | 0.05 |  |
|  | **24 h post-EIMD** | | | | |
|  | Single | -1.77 | -3.77 to 0.24 | 0.08 | Q = 5.7, p = 0.17 |
|  | Double | -0.276 | -0.99 to 0.46 | 0.48 |  |
|  | **48 h post-EIMD** | | | | |
|  | Single | -0.99 | -2.62 to 0.65 | 0.24 | Q = 2.92, p = 0.23 |
|  | Double | -0.63 | -1.63 to 0.37 | 0.22 |  |
| **Training status** | **Immediately post-EIMD** | | | | |
|  | Trained | -0.35 | -0.85 to 0.16 | 0.18 | Q = 7.63, p = 0.02 |
|  | Untrained | -0.5 | -0.91 to -0.09 | 0.02 |  |
|  | **24 h post-EIMD** | | | | |
|  | Trained | -0.81 | -1.74 to 0.12 | 0.09 | Q = 3.04, p = 0.22 |
|  | Untrained | -0.25 | -1.71 to 1.21 | 0.74 |  |
|  | **48 h post-EIMD** | | | | |
|  | Trained | -0.61 | -1.77 to 0.55 | 0.31 | Q = 4.97, p = 0.8 |
|  | Untrained | -0.93 | -1.86 to -0.01 | 0.05 |  |
| **Sex** | **Immediately post-EIMD** | | | | |
|  | Male | -0.62 | -1.03 to -0.22 | 0.003 | Q = 9.38, p = 0.02 |
|  | Female | -0.18 | -0.77 to 0.41 | 0.55 |  |
|  | Male and Female | -0.1 | -1.08 to 0.88 | 0.85 |  |
|  | **24 h post-EIMD** | | | | |
|  | Male | -1.11 | -1.81 to -0.41 | 0.002 | Q = 15.34, p = 0.002 |
|  | Female | 0.71 | -1.02 to 2.24 | 0.42 |  |
|  | Male and Female | 1.23 | 0.16 to 2.29 | 0.02 |  |
|  | **48 h post-EIMD** | | | | |
|  | Male | -1.05 | -1.75 to -0.35 | 0.003 | Q = 20.81, p < 0.01 |
|  | Female | -0.4 | -1.21 to 0.41 | 0.71 |  |
|  | Male and Female | 2.11 | 0.89 to 3.33 | 0.001 |  |

**Table S4** Subgroup analyses of categorical variables on DOMS at Immediately, 24 h, and 48 h post-EIMD

| **Follow-up time point**  Subgroup | | **ES** | **95% CI** | **p** | **Test of Moderators** |
| --- | --- | --- | --- | --- | --- |
| **Study design** | **Immediately post-EIMD** | | | | |
|  | Crossover | -0.88 | -1.75 to 0.002 | 0.05 | Q = 3.97, p = 0.14 |
|  | Parallel | -0.11 | -0.67 to 0.45 | 0.71 |  |
|  | **24 h post-EIMD** | | | | |
|  | Crossover | -1.08 | -1.72 to -0.45 | 0.001 | Q = 22.51, p < 0.001 |
|  | Parallel | -1.44 | -2.28 to -0.6 | 0.001 |  |
|  | **48 h post-EIMD** | | | | |
|  | Crossover | -0.88 | -1.75 to 0.002 | 0.05 | Q = 15.23, p < 0.001 |
|  | Parallel | -2.03 | -3.2 to -0.85 | 0.001 |  |
| **Blinding** | **Immediately post-EIMD** | | | | |
|  | Single | -0.77 | -1.51 to -0.04 | 0.04 | Q = 4.3, p = 0.12 |
|  | Double | 0.03 | -0.54 to 0.6 | 0.92 |  |
|  | **24 h post-EIMD** | | | | |
|  | Single | -1.77 | -2.64 to -0.9 | 0.001 | Q = 22.95, p < 0.001 |
|  | Double | -1.03 | -1.8 to 0.27 | 0.008 |  |
|  | **48 h post-EIMD** | | | | |
|  | Single | -2.39 | -3.96 to -0.82 | 0.003 | Q = 14.54, p = 0.002 |
|  | Double | -1.43 | -2.61 to -0.25 | 0.02 |  |
| **Training status** | **Immediately post-EIMD** | | | | |
|  | Trained | -0.37 | -1.16 to 0.41 | 0.35 | Q = 1.79, p = 0.41 |
|  | Untrained | -0.25 | -0.77 to 0.26 | 0.34 |  |
|  | **24 h post-EIMD** | | | | |
|  | Trained | -1.16 | -2.03 to -0.28 | 0.009 | Q = 18.51, p < 0.01 |
|  | Untrained | -1.52 | -2.38 to -0.65 | 0.001 |  |
|  | **48 h post-EIMD** | | | | |
|  | Trained | -2.01 | -3.62 to -0.41 | 0.014 | Q = 11.89, p = 0.01 |
|  | Untrained | -1.57 | -2.85 to 0.3 | 0.02 |  |
| **Sex** | **Immediately post-EIMD** | | | | |
|  | Male | -0.57 | -1.04 to -0.11 | 0.02 | Q = 6.71, p = 0.035 |
|  | Male and Female | -0.34 | -0.41 to 1.09 | 0.37 |  |
|  | **24 h post-EIMD** | | | | |
|  | Male | -1.56 | -2.22 to -0.89 | 0.001 | Q = 25.6, p < 0.001 |
|  | Female | -0.9 | -1.74 to -0.06 | 0.04 |  |
|  | Male and Female | -0.14 | -1.12 to 0.84 | 0.78 |  |
|  | **48 h post-EIMD** | | | | |
|  | Male | -2.19 | -3.22 to -1.16 | 0.001 | Q = 18.97, p < 0.001 |
|  | Female | -0.48 | -1.29 to 0.33 | 0.24 |  |
|  | Male and Female | -0.2 | -1.18 to 0.78 | 0.69 |  |

**Table S5** The SIGN RCT checklist for each included study

| **Study** | **1.1** | **1.2** | **1.3** | **1.4** | **1.5** | **1.6** | **1.7** | **1.8** | **1.9** | **1.10** | **2.1** |
| --- | --- | --- | --- | --- | --- | --- | --- | --- | --- | --- | --- |
| [Amirsasan et al. [40]](#_ENREF_40) | Y | Y | N | Y | Y | Y | Y | 0 | Y | CS | **++** |
| [Areces et al. [63]](#_ENREF_63) | Y | Y | N | Y | Y | Y | Y | 0 | Y | CS | **++** |
| [Atashak and Baturak [64]](#_ENREF_64) | Y | Y | N | Y | Y | Y | Y | 0 | Y | CS | **++** |
| [Barzegari [65]](#_ENREF_65) | Y | Y | N | Y | Y | Y | Y | 0 | Y | CS | **++** |
| [Dorrell and Gee [41]](#_ENREF_41) | Y | Y | N | Y | Y | Y | Y | 0 | Y | CS | **++** |
| [Gee and Deniel [58]](#_ENREF_58) | Y | Y | N | Y | Y | Y | Y | 0 | Y | CS | **++** |
| [Greer et al. [59]](#_ENREF_59) | Y | CS | N | Y | Y | Y | Y | 0 | Y | CS | **+** |
| [Howatson et al. [66]](#_ENREF_66) | Y | Y | N | Y | Y | Y | Y | 0 | Y | CS | **++** |
| [Jackman et al. [67]](#_ENREF_67) | Y | CS | N | Y | Y | Y | Y | 0 | Y | CS | **++** |
| [Kim et al. [68]](#_ENREF_68) | Y | Y | N | Y | N | Y | Y | 4 | Y | CS | **+** |
| [Koba et al. [55]](#_ENREF_55) | Y | CS | N | Y | Y | Y | Y | 0 | Y | CS | **+** |
| [Koo et al. [60]](#_ENREF_60) | Y | CS | N | Y | Y | Y | Y | 0 | Y | CS | **+** |
| [Ra et al. [56]](#_ENREF_56) | Y | Y | N | Y | Y | Y | Y | 0 | Y | CS | **++** |
| [Sheikholeslami-Vatani and Ahmadi [61]](#_ENREF_61) | Y | Y | N | Y | Y | Y | Y | 0 | Y | CS | **++** |
| [Shenoy et al. [69]](#_ENREF_69) | Y | Y | N | Y | Y | Y | Y | 0 | Y | CS | **++** |
| [Shimomura et al. [62]](#_ENREF_62) | Y | CS | N | Y | Y | Y | Y | 0 | Y | CS | **+** |
| [VanDusseldorp et al. [57]](#_ENREF_57) | Y | Y | N | Y | Y | Y | Y | 0 | Y | CS | **++** |
| [Waldron et al. [70]](#_ENREF_70) | Y | Y | N | Y | Y | Y | Y | 0 | Y | CS | **++** |

1.1: Focused Question; 1.2: Randomized Groups; 1.3: Concealment; 1.4: Blinded Allocation; 1.5: Similar Groups; 1.6: Treatment; 1.7: Outcomes; 1.8: Drop Out; 1.9: Analysis; 1.10: Comparable Results; 2.1: Final Ranking; Y: Yes; CS: Can’t Say; N: No; ++: High Quality; +: Acceptable.

**Fig. S1** Regression of hedges’ g on total dosage of BCAA of CK at 48 h post-EIMD

**Fig. S2** Regression of Hedges’ g on supplementation period of CK at 48 h post-EIMD.

**Fig. S3** Funnel plots of CK levels at (A) immediately, (B) 24 h, (C) 48 h, (D) 72 h, and (E) 96 h post-EIMD.

**Fig. S4** Sensitivity analysis showing reliability and stability of CK levels at immediately post-EIMD.

**Fig. S5** Sensitivity analysis showing reliability and stability of CK levels at 24 h post-EIMD.

**Fig. S6** Sensitivity analysis showing reliability and stability of CK levels at 48 h post-EIMD.

**Fig. S7** Sensitivity analysis showing reliability and stability of CK levels at 72 h post-EIMD.

**Fig. S8** Sensitivity analysis showing reliability and stability of CK levels at 72 h post-EIMD.

**Fig. S9** Funnel plots of LDH levels at (A) immediately, (B) 24 h, and (C) 48 h post-EIMD.

**Fig. S10** Sensitivity analysis showing reliability and stability of LDH levels at immediately post-EIMD.

**Fig. S11** Sensitivity analysis showing reliability and stability of LDH levels at 24 h post-EIMD.

**Fig. S12** Sensitivity analysis showing reliability and stability of LDH levels at 48 h post-EIMD.

**Fig. S13** Regression of Hedges’ g on daily dosage of DOMS at 24 h post-EIMD.

**Fig. S14** Funnel plots of DOMS at (A) immediately, (B) 24 h, (C) 48 h, (D) 72 h, and (E) 96 h post-EIMD.

**Fig. S15** Sensitivity analysis showing reliability and stability of DOMS levels at immediately post-EIMD.

**Fig. S16** Sensitivity analysis showing reliability and stability of DOMS levels at 24 h post-EIMD.

**Fig. S17** Sensitivity analysis showing reliability and stability of DOMS levels at 48 h post-EIMD.

**Fig. S18** Sensitivity analysis showing reliability and stability of DOMS levels at 72 h post-EIMD.

**Fig. S19** Sensitivity analysis showing reliability and stability of DOMS levels at 96 h post-EIMD.

**Table S2** PRISMA checklist.

| **Section and Topic** | **Item #** | **Checklist item** | **Location where item is reported** |
| --- | --- | --- | --- |
| **TITLE** | | |  |
| Title | 1 | Identify the report as a systematic review. | Page 1 |
| **ABSTRACT** | | |  |
| Abstract | 2 | See the PRISMA 2020 for Abstracts checklist. | Page 3 |
| **INTRODUCTION** | | |  |
| Rationale | 3 | Describe the rationale for the review in the context of existing knowledge. | Pages 4-5 |
| Objectives | 4 | Provide an explicit statement of the objective(s) or question(s) the review addresses. | Page 6 |
| **METHODS** | | |  |
| Eligibility criteria | 5 | Specify the inclusion and exclusion criteria for the review and how studies were grouped for the syntheses. | Page 6 |
| Information sources | 6 | Specify all databases, registers, websites, organisations, reference lists and other sources searched or consulted to identify studies. Specify the date when each source was last searched or consulted. | Page 6 |
| Search strategy | 7 | Present the full search strategies for all databases, registers and websites, including any filters and limits used. | Page 6 |
| Selection process | 8 | Specify the methods used to decide whether a study met the inclusion criteria of the review, including how many reviewers screened each record and each report retrieved, whether they worked independently, and if applicable, details of automation tools used in the process. | Pages 6-7 |
| Data collection process | 9 | Specify the methods used to collect data from reports, including how many reviewers collected data from each report, whether they worked independently, any processes for obtaining or confirming data from study investigators, and if applicable, details of automation tools used in the process. | Page 7 |
| Data items | 10a | List and define all outcomes for which data were sought. Specify whether all results that were compatible with each outcome domain in each study were sought (e.g. for all measures, time points, analyses), and if not, the methods used to decide which results to collect. | NA |
|  | 10b | List and define all other variables for which data were sought (e.g. participant and intervention characteristics, funding sources). Describe any assumptions made about any missing or unclear information. | NA |
| Study risk of bias assessment | 11 | Specify the methods used to assess risk of bias in the included studies, including details of the tool(s) used, how many reviewers assessed each study and whether they worked independently, and if applicable, details of automation tools used in the process. | Page 7 |
| Effect measures | 12 | Specify for each outcome the effect measure(s) (e.g. risk ratio, mean difference) used in the synthesis or presentation of results. | Pages 7-8 |
| Synthesis methods | 13a | Describe the processes used to decide which studies were eligible for each synthesis (e.g. tabulating the study intervention characteristics and comparing against the planned groups for each synthesis (item #5)). | Pages 7-8 |
|  | 13b | Describe any methods required to prepare the data for presentation or synthesis, such as handling of missing summary statistics, or data conversions. | Pages 7-8 |
|  | 13c | Describe any methods used to tabulate or visually display results of individual studies and syntheses. | Pages 7-8 |
|  | 13d | Describe any methods used to synthesize results and provide a rationale for the choice(s). If meta-analysis was performed, describe the model(s), method(s) to identify the presence and extent of statistical heterogeneity, and software package(s) used. | Pages 7-8 |
|  | 13e | Describe any methods used to explore possible causes of heterogeneity among study results (e.g. subgroup analysis, meta-regression). | Pages 7-8 |
|  | 13f | Describe any sensitivity analyses conducted to assess robustness of the synthesized results. | Pages 7-8 |
| Reporting bias assessment | 14 | Describe any methods used to assess risk of bias due to missing results in a synthesis (arising from reporting biases). | Pages 7 |
| Certainty assessment | 15 | Describe any methods used to assess certainty (or confidence) in the body of evidence for an outcome. | NA |
| **RESULTS** | | |  |
| Study selection | 16a | Describe the results of the search and selection process, from the number of records identified in the search to the number of studies included in the review, ideally using a flow diagram. | Page 9 |
|  | 16b | Cite studies that might appear to meet the inclusion criteria, but which were excluded, and explain why they were excluded. | Page 9 |
| Study characteristics | 17 | Cite each included study and present its characteristics. | Page 9 |
| Risk of bias in studies | 18 | Present assessments of risk of bias for each included study. | Page 12 |
| Results of individual studies | 19 | For all outcomes, present, for each study: (a) summary statistics for each group (where appropriate) and (b) an effect estimate and its precision (e.g. confidence/credible interval), ideally using structured tables or plots. | Pages 9-12 |
| Results of syntheses | 20a | For each synthesis, briefly summarise the characteristics and risk of bias among contributing studies. | Pages 9-12 |
|  | 20b | Present results of all statistical syntheses conducted. If meta-analysis was done, present for each the summary estimate and its precision (e.g. confidence/credible interval) and measures of statistical heterogeneity. If comparing groups, describe the direction of the effect. | Pages 9-12 |
|  | 20c | Present results of all investigations of possible causes of heterogeneity among study results. | Pages 9-12 |
|  | 20d | Present results of all sensitivity analyses conducted to assess the robustness of the synthesized results. | Pages 9-12 |
| Reporting biases | 21 | Present assessments of risk of bias due to missing results (arising from reporting biases) for each synthesis assessed. | Pages 9-12 |
| Certainty of evidence | 22 | Present assessments of certainty (or confidence) in the body of evidence for each outcome assessed. | NA |
| **DISCUSSION** | | |  |
| Discussion | 23a | Provide a general interpretation of the results in the context of other evidence. | Pages 13-17 |
|  | 23b | Discuss any limitations of the evidence included in the review. | Pages 13-17 |
|  | 23c | Discuss any limitations of the review processes used. | Page 17 |
|  | 23d | Discuss implications of the results for practice, policy, and future research. | Page 17 |
| **OTHER INFORMATION** | | |  |
| Registration and protocol | 24a | Provide registration information for the review, including register name and registration number, or state that the review was not registered. | NA |
|  | 24b | Indicate where the review protocol can be accessed, or state that a protocol was not prepared. | NA |
|  | 24c | Describe and explain any amendments to information provided at registration or in the protocol. | NA |
| Support | 25 | Describe sources of financial or non-financial support for the review, and the role of the funders or sponsors in the review. | Page 19 |
| Competing interests | 26 | Declare any competing interests of review authors. | Page 19 |
| Availability of data, code and other materials | 27 | Report which of the following are publicly available and where they can be found: template data collection forms; data extracted from included studies; data used for all analyses; analytic code; any other materials used in the review. | NA |
